# Supplementary material for: Translating concerns into action: a detailed qualitative evaluation of an interdisciplinary intervention on medical wards
Source: BMJ Open. 2017 Apr 5;7(4):e014401. doi: 10.1136/bmjopen-2016-014401 (PMC5719651; doi:10.1136/bmjopen-2016-014401)
Supplement: Author's manuscript [file bmjopen-2016-014401.draft_revisions.pdf]

BMJ Open

Translating concerns into action: a detailed qualitative evaluation of an interdisciplinary intervention on medical wards

Journal:	*BMJ Open*
Manuscript ID	bmjopen-2016-014401
Article Type:	Research
Date Submitted by the Author:	22-Sep-2016
Complete List of Authors:	Pannick, Samuel; Imperial College London, NIHR Imperial Patient Safety Translational Research Centre<br>Archer, Stephanie; Imperial College London, NIHR Imperial Patient Safety Translational Research Centre<br>Beveridge, Iain; West Middlesex University Hospital NHS Trust, Department of Medicine<br>Long, Susannah; Imperial College London, Centre for Patient Safety and Service Quality<br>Athanasiou, Thanos; Imperial College London, Surgery and Cancer<br>Sevdalis, Nick; King's College London,
**Primary Subject Heading**:	Health services research
Secondary Subject Heading:	Health services research, Medical management, Qualitative research
Keywords:	Quality in health care < HEALTH SERVICES ADMINISTRATION & MANAGEMENT, interdisciplinary teams, medical ward, prospective surveillance

SCHOLARONE™ Manuscripts

For peer review only

Translating concerns into action: a detailed qualitative evaluation of an interdisciplinary intervention on medical wards

Samuel Pannick,[1] Stephanie Archer,[1] Iain Beveridge,[2]
Susannah J Long,[1,3] Thanos Athanasiou,[4] & Nick Sevdalis[5]

[1] NIHR Imperial Patient Safety Translational Research Centre, Imperial College London
[2] West Middlesex University Hospital, Isleworth, UK
[3] Imperial College Healthcare NHS Trust, London, UK
[4] Department of Surgery & Cancer, Imperial College London, UK
[5] Centre for Implementation Science, King's College London

Corresponding author:
Dr Sam Pannick, MA MB BS MRCP
Room 503 Medical School Building, Norfolk Place
London W2 1NY
United Kingdom
s.pannick@imperial.ac.uk; + (44) 207 3543149

Key words: patient safety; healthcare quality; interdisciplinary teams; medical ward; prospective surveillance

Abstract length: 278 words

Manuscript length: 5584 words

Online content: Box 1

For peer review only

ABSTRACT

Objectives

To understand how frontline reports of day-to-day care failings might be better translated into improvement.

Design

Qualitative evaluation of an interdisciplinary team intervention to capitalise on the frontline experience of care delivery. Prospective clinical team surveillance (PCTS) involved structured interdisciplinary briefings to capture challenges in care delivery, facilitated organisational escalation of the issues they identified, and feedback. 18 months of ethnography and two focus groups were conducted with staff taking part in a trial of PCTS.

Results

PCTS fostered psychological safety – a confidence that the team would not embarrass or punish those who speak up. This was complemented by a hard edge of accountability, whereby team members would regulate their own behaviour in anticipation of future briefings. Frontline concerns were triaged to managers, or resolved autonomously by ward teams, reversing what had been well-established normalisations of deviance. Junior clinicians found a degree of catharsis in airing their concerns, and their teams became more proactive in addressing improvement opportunities. PCTS generated tangible organisational changes, and enabled managers to make a convincing case for investment. Briefings were constrained by the need to preserve professional credibility, and the relative comfort afforded by the avoidance of accountability. At higher organisational levels, frontline concerns were subject to competition with other priorities, and their resolution was limited by the scale of the challenges they described.

For peer review only

Conclusions

Prospective safety strategies relying on staff-volunteered data do approximate the realities of frontline care, but still produce acceptable, negotiated accounts, subject to the many interdisciplinary tensions that characterise ward work. Nonetheless, they give managers access to these accounts, and support frontline staff to make incremental changes in their daily work. These are goals for learning healthcare organisations.

Trial registration

ISRCTN 34806867

STRENGTHS AND LIMITATIONS OF THIS STUDY

Qualitative evaluations of complex interventions are recommended by the Medical Research Council.

This study identified the mechanisms to be replicated if further PCTS implementation efforts are to prove successful.

The evaluation highlighted tensions within efforts to capitalise on the frontline experience of care delivery; previous reports had characterised frontline data collection as a more objective exercise.

Selection bias may reduce the internal validity of our findings: there was disproportionate access to staff who engaged with the intervention.

As a single-site study, the generalisability of the results may be limited.

For peer review only

INTRODUCTION

Patient safety – the absence of preventable harm to a patient during the process of healthcare – is now considered a serious global public health issue.[1] Around 10% of in-patients are still affected by preventable harms.[2,3] Many of those harms are the result of persistent organisational failings, either deliberately ignored, or to which the organisation has become blind.[4] This study seeks to understand how healthcare organisations can better identify and resolve problems in the delivery of care. It does so through a detailed qualitative evaluation of an interdisciplinary intervention, which captured and disseminated the daily challenges of frontline staff as they provided care.

Frontline staff have a particular insight into the reality of failings in day-to-day care, which are not necessarily captured in high-level aggregated organisational metrics.[5] Frontline views differ to those in senior leadership positions, and are valuable for that:[6] they may challenge longstanding assumptions about organisational performance. Accessing and interpreting these frontline views, to go beyond conventional metrics, represents 'soft intelligence' – revealing discomfiting, potentially important, institutional fallibilities.[7] Senior healthcare leaders readily acknowledge the importance of soft data,[8] and those that produce soft intelligence may generate more holistic improvement strategies as a result.[7] Yet those same leaders are wary of expending effort and resources in pursuing concerns that might ultimately prove to be trivial, and look for ways to validate frontline reports before putting them to use.[8] How this paradox can be managed best has not yet been investigated.

A separate literature has focused on the other end of the leadership hierarchy, examining how frontline teams identify and manage safety and quality concerns in the course of their normal work. That they do so is relatively clear.[9-12] Yet frontline staff do not typically volunteer information about the problems they encounter: from an organisational viewpoint, the problems remain hidden. Whilst operational issues are commonplace, the vast majority are immediately addressed at the

For peer review only

frontline, the staff member finding a way to compensate and complete the task that had been blocked or interrupted. This has been described as *first-order problem solving*, i.e., generating short-term remedies that are adequate in that moment but do nothing to prevent the problem's recurrence.[13] This first-order problem solving is encouraged by professional pride,[14] an organisational emphasis on individual vigilance, and by the premium that managers place on frontline troubleshooting.[13,15] *Second-order problem solving* – which is much rarer – requires additional escalation, communication or follow-up to address the underlying causes of the problem, and facilitates organisational improvement.[13] To date, neither local managers nor national safety campaigns have focused on this frontline-driven improvement,[16,17] despite calls from senior figures for it to be prioritised.[18,19]

Here, we focus on the translation of frontline concerns into tangible system improvement, in the setting of general medical ward care. Medical wards deliver the majority of acute inpatient care in health systems worldwide, and care for a particularly heterogeneous group of patients, facing a combination of challenges not shared by other clinical microsystems.[20] These wards contribute disproportionately to preventable hospital deaths,[21] but still labour to attract organisational attention and resources.[16] As a result, medical ward staff balance competing risks to patient safety, juggling priorities but also cutting corners.[10] In doing so, they are careful to protect their professional identities and credibility,[22] externalising blame if and when things do go wrong. Problems are frequently explained with reference to a lack of staffing and resources; the alternative – calling attention to individual failings – would breach important social norms.[10] Barriers to medical ward improvement therefore include the nature of the work, relationships with organisational leaders, team structures and norms, and individual priorities.

A novel intervention, prospective clinical team surveillance (PCTS), aimed to address these barriers and capitalise on medical ward teams' daily experiences. PCTS combines structured interdisciplinary

For peer review only

briefings to capture frontline clinical and administrative challenges, with facilitated organisational escalation of the issues they identified, and feedback. The structured briefings reviewed the events of the previous shift, identifying systemic problems and patients who had deteriorated, and concluded with a team plan to resolve or escalate those concerns. After a short period of supervision, ward teams led their own briefings autonomously. A single facilitator helped teams advance the issues raised in the briefings, and provided follow-up and feedback to stakeholders throughout the organisation. Briefings were known locally as 'HEADS-UP briefings'. The intervention is described in more detail in the published protocol.[23] Implemented alongside existing incident reporting systems between 2013 and 2015, PCTS was evaluated in a pragmatic, stepped wedge, cluster controlled trial, involving seven medical ward teams from two London (UK) hospitals. Compared to usual care, high fidelity PCTS reduced excess length of stay, and improved teamwork and safety climates, and incident reporting.[24]

In this study, we seek to understand the effects of PCTS on frontline staff and managers, through the application of complementary qualitative methodologies, namely ethnography and focus groups. Our aim was to describe the intervention's effects as understood and enacted by these staff – individually, as teams, and in their relationships with professionals outside the immediate team.

METHODS

Process evaluation

The Medical Research Council (MRC) has emphasised the need for process evaluations of complex interventions like PCTS, to articulate the causal mechanisms by which the intervention produced change. The study was grounded in the recent iteration of the MRC framework.[25] Qualitative methods help to better identify complex causal pathways and unexpected mechanisms of impact; detailed understanding of these is vital, if successful quality improvement initiatives are to be

For peer review only

replicated elsewhere. Ethnography and focus groups are recommended as key tools for this type of investigation.[26]

Embedded research and auto-ethnography

The programme lead was an embedded researcher at the institution contributing six of the seven study wards. This position is defined as 'work[ing] inside [a] host organisation as [a] member of staff, while also maintaining an affiliation with an academic institution… collaborating with teams within the organisation to… conduct research studies… which respond to the needs of the organisation, and accord with its unique context and culture'.[27] Relationships between staff and the researcher are important, as the researcher is seen both to be part of the team and contributing to the research capacity of the host organisation.[28] Fieldnotes, typed contemporaneously as soon as was practicable, recorded personal exchanges and emails with staff, changes arising from PCTS, governance proceedings, reflections on the broader context, and other implementation challenges. These 'auto-ethnographic' insights have been widely used in organisational case study research, providing broad accounts of culture and practice.[29-31]

Focus groups

Semi-structured focus groups were undertaken in July 2015. Focus groups were appropriate as the topics of interest were specific attitudes, feelings and beliefs that would best be revealed through social interactions between group participants.[32] The topic guide explored PCTS as a tool, as well as the implementation process that introduced it. Participants were asked to discuss existing systems for identifying team concerns and improving patient care, to orientate themselves to the topic. They then reflected on their experiences of using PCTS. Questions and follow-up probes scrutinised team-wide involvement in PCTS; whether it affected perceptions of the ward's safety and quality; how it was used in staff interactions; and whether changes had been made as a result. Topic guide questions were reviewed and piloted within the research team, and with researchers in healthcare

For peer review only

quality and safety who had not been involved with the study previously. The focus groups were advertised in clinical areas, by email, and in person; all ward staff were invited to attend. Certain staff groups were purposively targeted to ensure that key stakeholders in generating and processing the frontline briefing data (junior doctors, senior nurses, and service managers) were represented. Focus groups lasted approximately two hours, each facilitated by two experienced qualitative researchers, using the topic guide flexibly in view of the different roles and experiences within the two groups. Focus group discussions were digitally recorded, and then professionally transcribed.

Focus group transcripts and fieldnotes were managed with NVivo software (QSR International, Doncaster, Australia). Two researchers read and re-read the source material, adopting an inductive (theory-generating) approach.[33] Each researcher coded transcripts individually, generating an initial coding frame, which was then discussed and refined. The transcripts were coded again, before a group of higher order themes was agreed. Field notes were searched for evidence to support or contradict the evidence from the focus groups, and for themes not covered independently by focus group discussions. Themes were inspected against the broader literature to inform their interpretation. The study was approved by research and development authorities at participating institutions as a quality improvement programme. Focus group participants gave their signed consent. The primary study was registered with the ISRCTN registry (ISRCTN34806867).

RESULTS

The two semi-structured focus groups included 15 staff: three junior doctors (foundation doctors, in their first two years of medical practice), eight senior ward nurses and four non-clinical managers. Focus group data were supplemented by 44 pages of auto-ethnographic fieldnotes, documenting an 18-month period in which links were established with clinicians; hybrid managers with clinical and managerial workloads;[34,35] middle managers, who reported to the executive team; and senior hospital leaders.

For peer review only

Our findings are organised around four themes. First, we describe the shared team beliefs and ethos established by PCTS. Second, we discuss how teams used the programme to triage ward problems more effectively. Third, we identify how individuals' practice changed as a result of their participation. Finally, we note how PCTS altered team and organisational approaches to improvement, and the tangible changes that came about as a result.

1. PCTS established psychological safety, with a hard edge of accountability

The briefings were the first structured tool the study teams had used for their routine interdisciplinary work. Still, by the end of the study period, all staff groups had participated in – and indeed led – the briefings:

> '[Consultants] contribute as much as anyone else […] so their voice is being heard, because they suffer from the same frustrations as all of us.' *(Service manager)*

> 'The physio[therapists] or the OTs [occupational therapists] are also flagging things up, that's how we found it useful. […] The discharges that did not happen, anyone [who] deteriorated, they flag it up and then we all learn from what went wrong.' *(Senior nurse)*

Perhaps because the different staff groups were perceived as partners in them, the HEADS-UP briefings formed a psychologically safe environment in which problems could be discussed openly, without fear of retribution. Team psychological safety does not imply undue permissiveness or unrelenting cheeriness, but a confidence that the team will not embarrass or punish someone for speaking up (Edmondson, 1999; Tucker, 2007):

For peer review only

> ‘It was more of a constructive exercise, where everyone is then wanting the same outcome, so it wasn’t like, “Oh this didn’t happen, I am angry at you”, it was more like we are all actually in the same team and we all want the same thing to happen… You all have the same end point and I think that is why it is quite safe to bring it up, because it is not confrontational, and it is not personal against someone, it is just what needs to be done.’ *(Foundation doctor)*

The style and timeliness of PCTS feedback also contributed to a sense that this was a non-judgemental forum for team learning:

> ‘There were a few teaching sessions where things were raised, we went through things like ECGS [electrocardiographs] that were missed, and then you had a collective opportunity to think. […] It was a very good plot for learning, saying this happened and let’s do some learning and try and prevent it happening again. That is quite helpful.’ *(Foundation doctor)*

This psychological safety was not limitless: there were still boundaries around what could be discussed. Although the briefings did prompt teams to consider positive notes from the previous shift, they rarely did so. When they did record something positive, their reflections often concerned the overall management of patient flow, rather than specific actions or diagnostic processes that others could emulate:

> ‘Maybe it’s just I am also still learning to flag up what went well the day before, because we are all good with, “Ah, this was frustrating yesterday…”’ *(Senior nurse)*

This reflected a reluctance to delve too deeply into any one team member’s performance. Professional identities were protected by assigning problems to other departments, acknowledging

For peer review only

procedural complications as foreseeable, or linking delays to under-staffing. Thus, perceived threats to professional identity were largely deflected by a projection of blame onto other groups, or attributed to circumstances beyond the team's control.[36,37] More introspective teams, with senior support and increasing psychological safety, did record issues like diagnostic errors, in which they had played a more overt role. However, even in those teams – where the briefings were implemented with greatest fidelity – there could be disputes as to whether certain problems were really problems at all:

> '[The senior nurse] disagrees with medical teams' perceptions that the site moves [moving patients from one ward to another] were inappropriate. She thinks doctors expect patients to remain on the acute medical unit even when they are stable… She felt an inadequate handover perpetuated their concerns.' *(Fieldnotes)*

Thus, whilst psychological safety was an important factor in helping staff speak up about their concerns, certain topics remained off-limits, and psychological safety did not guarantee agreement about what were reasonable concerns. These are important limitations.

The briefings also maintained a 'hard edge' for their effectiveness.[38] Team members regulated their own behaviour, knowing that their actions (or inactions) might be flagged up at the next briefing, or in feedback. Internalising discipline in this way (panopticism) plays an important part in quality and safety governance in English hospitals.[39] Junior doctors and nurses were conscious of this panopticism, acknowledging how it changed their own behaviour and how they could use it to their advantage:

For peer review only

'The group was more aware that if you perhaps missed something like that, it may be brought up later at a HEADS-UP meeting. […] It made you more accountable.' *(Foundation doctor)*

'It certainly works for the VTEs [venous thromboembolism assessments, completed by junior doctors, often at the behest of nursing staff]. There are days that we will have eight or 10 VTEs [to do], so if I […] give the list to the doctor, the following day […] there will be one or two, because they know […] it will be mentioned again, with the consultant.' *(Senior nurse)*

Although the briefings emphasised accountability, the teams did not always explicitly agree exactly what would be done, and by when, as seen previously in interdisciplinary initiatives.[40] This supports the idea that teams may actually find comfort in systems that allow them to avoid accountability.[10] Shift patterns that lacked continuity exacerbated this problem, if briefing leaders neglected to confirm whether any issues were still outstanding:

'I think it's more [than] just raising the issue. […] Give me a more detailed timeframe, resolution, how do you resolve it? Are you going to get back to me because it's important? And what will I expect?' *(Senior nurse)*

Others had more positive experiences where, held to account, their colleagues confirmed the outcomes of previous briefings:

'You get more feedback, so you raise concern[s], and then maybe the next day the same issue would crop up, and you actually then get feedback on what they are doing about it, or who they have escalated to.' *(Foundation doctor)*

For peer review only

As we will now describe, airing and recording concerns in this environment helped triage those problems, directing them back to frontline teams or onwards to their managers.

2. Rapid resolution and meaningful managerial follow-up

To explain their perceptions of PCTS, focus group participants drew comparisons with the existing processes for identifying and resolving ward-level problems. The hospital's online incident reporting system was considered the formal mechanism by which problems were recorded. The system was not easy to use for this purpose, and there were few attempts to persistently report recurrent problems. As a result, commonplace issues were no longer even considered abnormal – a materialisation of normalised deviance, where staff become so desensitised to deviant practice that it no longer feels wrong:[41]

> 'Some things staff have got so used to that they don't [report] it. So it's just become common practice.' *(Clinical risk manager)*

Managers too were frustrated by the online incident reporting system, which they had little time for, and did not find useful:

> 'You could go down there [to the ward] and you could see people working very, very hard and you could also sense a lot of frustrations […] but you weren't getting to what those frustrations were, and what those issues were.' *(Service manager)*

Long delays in processing incident reports meant that even relatively simple problems were not reviewed for many weeks. Even a broken piece of equipment could take '*weeks on end*'. As a result,

For peer review only

reports that described patient harm were prioritised; where no harm had occurred, little was done. Service managers were aware that they paid attention to only the most serious incidents:

> 'It has got to that point of being a SI [serious incident, an adverse event with particularly grave consequences] before it is then addressed and taught and learnt from.' *(Service manager)*

In contrast, PCTS helped identify a route for more rapid resolution of practical problems. It provided an acceptable mechanism for staff to log issues into which they had immediate insight:

> '[PCTS] highlighted little things, that could be sorted out quite quickly. It made sure that somebody was allocated to deal with that on the day – or did you follow that through, did that get sorted out? So everything benefits, rather than just going on and on and on […] Things get sorted out quicker.' *(Senior nurse)*

This was a materialisation of second-order problem solving, staff finding a way to bring about a more lasting solution to each issue they encountered. Where issues could not be resolved within the ward team, managers were presented with clear, actionable topics to address. They were then better prepared to handle incipient risks to service quality:

> 'What I like about it is [that] it is instant and it is thematic very quickly, you understand what the issues are […] Here is an opportunity to dump them [your daily issues] and suddenly a picture forms out of it […] And not just here, but there, there, there and there […] And it hasn't yet created a significant incident, but clearly there is risk associated with it.' *(Service manager)*

For peer review only

Not all managers were equally enthused. Where PCTS had not provided data that appeared directly useful for them, middle managers did less to hold their service areas accountable for briefing implementation. Still, the combination of increased frontline efficiency and managerial involvement (at least in some areas) led to a more general sense that things had changed for the better:

> '[PCTS] would make it clear what was actually deficient. And then I think things did start to change, and you did see the feedback coming through […] So you could definitely see the evolution of it.' *(Foundation doctor)*

> 'There is also the kind of day-to-day things within the team that start running better, and I think you do start seeing those, but it is harder to put a specific, "This was raised, this was done, there was an outcome." It is a more […] general change as part of [PCTS].' *(Foundation doctor)*

With a structure in place to coordinate frontline teams and their managers, individual staff found their own practice changing, with unexpected personal benefits. These will now be discussed in more detail.

3. Changing individuals: reversing normalised deviance, and catharsis

PCTS prompted staff to proactively address issues they may have previously ignored. This was, in effect, a reversal of the normalised deviance that had become so ingrained:

> 'You wouldn't have identified that [issue] necessarily, because it is just part of your daily […] life in the NHS, but when it is put like this [with PCTS], it is highlighted, you have got an opportunity to really do something about it.' *(Service manager)*

For peer review only

> 'This particular patient [has been] awaiting echo[cardiography] for the last three days […] [I] would now question why is it still not done […] When I wasn't doing [the briefings], I wasn't seeing the importance […] [Now] I tell the Matron, or I tell my Bed Manager […] or I go there myself […] I go to the department and ask them, "This is what's happening, this is what I need."' *(Senior nurse)*

Having made those problems visible once again, the information shared in the HEADS-UP briefings would be used to actively coordinate plans and decisions for the next shift. Aware of pressures on their colleagues, and cognisant that problems were occurring repeatedly, individuals would change their routines:

> 'I tell the physio[therapist] and the OT [occupational therapist]: "These are your priorities for today, these are your priorities for tomorrow." So they already have a plan […] Like I said, it gives you the structure that you need.' *(Senior nurse)*

> 'The group as a whole became more "present" to [what] happens maybe a couple of times in a week […] For example if the nursing staff are short […] just having that in your mind and thinking "No, we need to get all these blood tests done now", you would appreciate how the service could be best run. I think that made it more efficient.' *(Foundation doctor)*

The improved information sharing, interdisciplinary coordination, and efforts to evenly distribute workload produced a more supportive team climate. In this atmosphere, the briefings could become unexpectedly cathartic, giving a voice to junior clinicians to air their personal worries, as well as their clinical concerns:

For peer review only

'You felt like you had got a lot of your chest by doing it, and if you had a bad day and nothing seemed to be going right because of so many errors that you came across, it all felt a bit chaotic and scatty, the next day you could say, "These are the things that went wrong yesterday", and now I can clear that and start again.' *(Foundation doctor)*

'[It] makes you feel more heard, because otherwise you are just venting your concern, but you don't know if it has been actioned in any way. And then you can provide the objective evidence to move forward, to change something.' *(Foundation doctor)*

The combination of changes in team ethos, triage, and individual practice ultimately provoked a new approach to improvement, and a degree of organisational development.

4. Changing teams and the organisation

Contrary to some leaders' fears, PCTS did not reduce the volume of formal incident reports. Rather, it provoked new reports, and altered how those reports were addressed. The quantitative analysis had shown how high fidelity implementation affected the patterns of incident reporting:[24] here too, staff recognised that some of the events discussed in the briefings needed to be logged as formal incidents. However, the translation of briefing-recorded incidents into online reports was imperfect, not least because the department had no agreed list of high priority events to be recorded, a common problem:[42,43]

'Post-intervention antibiotics had not been given […] The junior doctors had not seen the procedure report specifying they were needed. No incident report was done, even though [the registrar] specifically asked if one should be completed.' *(Fieldnotes)*

For peer review only

When incidents were reported, the briefings allowed incident investigators to start to collect relevant information. The problems that had provoked the incident were typically captured by the briefing, giving the investigator (often the nurse in charge) a head start in understanding what had happened:

> `The people involved are already there, they are already able to explain to you, "This is what actually happened from our end." […] It's easier for you to do your incident [investigation] report already because as the investigator you don't have to call everyone.' *(Senior nurse)*

The emphasis on clinical teams' accountability also led to a subtler change in incident management. Teams took responsibility for issues that were actually within their power to change, rather than awaiting permission or hoping that the problems would be resolved elsewhere:

> 'Very often before it gets to me, it has been dealt with and sorted out by the teams themselves […] With [briefings] and [incident reporting] together, there is a different approach: […] "We have discussed it, we have taken on the responsibility of this, we have done as much of this as we can, but here is the point where it has to be escalated and we want to make sure something is done about it, and this is our methodology for doing it." That is how it feels to me, anyway.' *(Service manager)*

At multiple levels within the organisation, it was clear that this programme had revealed hidden gaps in practice. PCTS proved useful even on wards where teams were already thought to be functioning well:

> `There is definitely time to do it, there is definitely a need to do it, but […] you only know that once you have done it.' *(Service manager)*

For peer review only

> 'I don't think we would have known, unless we had done it, that there was a gap there.' *(Senior nurse)*

Similarly, PCTS revealed service provision opportunities that had not previously reached the board's attention. In safety and governance meetings, PCTS findings highlighted new areas for improvement, many of which had not been addressed in incident reports or top-down initiatives. Those findings then enabled managers to make a convincing case for investment:

> 'It empowers me then as manager for that area to go forward with a business case with the evidence to say we need additional resource, this is the implications of it […] And it works, so we have an additional [cardiac] sonographer […] I think we saw very quickly a reduction in delays once that was sorted.' *(Service manager)*

A number of other specific, tangible changes were driven by PCTS during the study period [box 1]. These changes were described as 'quick wins' by senior managers, with clinicians agreeing that they were at least a relatively rapid organisational response. However, internal investment was not necessarily targeted specifically to participating teams. For example, expansion of the phlebotomy service (identified through PCTS as no longer meeting patient needs) primarily benefited non-participant wards, which were deemed by senior management to be more in need of support. Nor was progress guaranteed by repeated ward-level reporting. Changes to structures or processes were more likely when there was an associated financial target; another organisational incentive aligning with the proposed improvement; and when clinicians and managers agreed the need for change.[44] Additionally, some of the concerns raised by frontline staff simply could not be addressed by middle managers, general managers or even board members. Poor staffing levels, for example, were felt keenly, but reflected a broader national challenge. Creative workarounds and mitigation plans could

only go so far in addressing a fundamental shortfall in staff. This was frustrating for the teams completing the briefings: the full scope of the challenges facing local improvement initiatives is often underestimated.[45]

DISCUSSION

We found that PCTS established a basis of team psychological safety, married with a hard edge of accountability, which in turn was generated by teams' own panopticism. Ward teams were able to triage recurrent problems to their managers, or to resolve them autonomously. In either case, PCTS – in making those challenges organisationally visible – fulfilled Tucker and Edmondson's criteria for second-order problem solving.[13] Through non-judgemental facilitation and feedback, ward teams reversed what had been well-established normalised deviance. Junior clinicians found a degree of catharsis, and teams became more proactive in addressing improvement opportunities. The soft intelligence provided through PCTS proved effective in generating tangible organisational change – albeit unpredictably, dependent on the hospital's other priorities and incentives. At the ward level, briefings were limited by the need to preserve professional credibility, a lack of interdisciplinary consensus about the nature of the problems they were seeing, and the relative comfort afforded by the avoidance of accountability. At higher organisational levels, frontline concerns were subject to competition with other priorities, and resolution was limited by the full scale of the challenges they described.

To our knowledge, this trial was the first controlled study of an interdisciplinary intervention to translate frontline experiences into organisational change. In turn, this report is the first qualitative analysis of how this type of interdisciplinary intervention might generate improved outcomes. Previous observational studies had shown how observers could work with ward staff to identify potential adverse events, building detailed assessments of ward performance in near-real time.[3,46,47] In these studies, frontline staff were the relatively passive objects of data collection: how they might

For peer review only

actively shape, frame, and mitigate reported safety concerns was not considered. Our current study therefore offers two novel contributions. First, it identifies the mechanisms that need to be replicated if other PCTS implementation efforts are to prove successful. Second, it highlights the innate tensions within these efforts to capitalise on the frontline experience of care delivery.

Frictions in organisational learning have been described previously. In reporting their concerns, staff portray 'others' as threatening patient safety, rather than jeopardising their own credibility.[22,48] Disputes about responsibility and appropriate care can stem from very real interdisciplinary disagreements about what the right course of action *should* have been,[49,50] and from divergent understandings of patient safety.[51] As concerns are escalated through an organisation, these important distinctions are often lost: rich narratives about patient safety events are repeatedly 'washed' of their context to better meet managerial assumptions.[48] The construction and escalation of a concern, therefore, remains a fundamentally subjective process. We found that our efforts to directly engage frontline teams in identifying their concerns did not eliminate these tensions. Indeed, frontline challenges are particularly likely to arise from informal rules, tacit agreements and intergroup conflicts,[50] and will remain disputed and contentious. Whilst junior clinicians are likely to embrace more flexible, engaging safety systems,[52] they may not anticipate these practical problems. Future efforts to involve frontline staff in prospective safety monitoring - whether through facilitated team self-report, as here, or with the help of embedded observers – should be mindful of these limitations.

Yet for all those complexities, staff described clear mechanisms by which PCTS improved team function and clinician-manager coordination, and generated organisational action. Frontline prospective surveillance strategies have been used in Canada[3,46] and the USA:[47] we have described a strategy that might be appropriate for the UK context. In demonstrating that their experiences have real organisational value, PCTS offers a route by which frontline staff (particularly junior staff) can be

For peer review only

'powerful agents for change',[18] and for leaders to obtain 'first-hand knowledge of the system at the frontline'.[19] We found that PCTS engaged transient, rotating, junior staff, whose views may be particularly insightful: they are not bound by the organisation's institutional narrative in the same way as their senior colleagues, who are more acclimatised to its norms.

Limitations of this study include our disproportionate access to the teams and individuals who engaged with PCTS, as opposed to those who declined to participate meaningfully. This was the case both for auto-ethnography and attendance at the focus groups. These enthusiasts may have had a more positive view of the programme than the wider group of eligible clinicians and managers. Selection bias has hampered mixed methods evaluations of other quality improvement interventions.[53] The high uptake of these interventions by teams that are already performing well, with less to improve, has also been noted previously.[54] We attempted to counteract any selection bias by ensuring the researchers leading the focus groups had not previously been directly involved in delivering the PCTS programme. We also conducted a deliberate search for conflicting opinions in the group transcripts and fieldnotes. In addition, our two methods were complementary. Any social desirability bias in the focus groups – participants giving the acceptable 'right' answers rather than revealing their true thoughts – was countered by the extensive ethnographic access to staff on the wards, in meetings with their peers, and in unguarded conversation. Our detailed evaluation may not generalise fully to other settings – although many of our findings built upon the existing understanding of frontline safety engagement.[43] Strengths of the study include the novel, theoretically-informed, qualitative analysis of a safety surveillance strategy that had previously been seen as purely objective. The embedded research model produced good access to managers as well as frontline staff; often, there is a trade-off between the two. The dual methodologies allowed the themes emerging from each data set to be triangulated, producing a more robust analysis than either one could in isolation. Importantly, the study identifies mechanisms by which frontline

For peer review only

concerns can be used productively, rather than disappearing into an organisational 'black hole'[14] from which they generate no change or learning.

CONCLUSIONS

PCTS, escalating frontline clinical and administrative challenges with facilitation and feedback, improved clinical outcomes through distinct mechanisms acting at the individual, team, and organisational levels. These mechanisms need to be replicated if other PCTS implementations are to improve outcomes in a similar fashion. Moreover, this detailed study demonstrates that prospective clinical surveillance strategies are not merely novel measurement tools, as they have been described[46,47,55] – even if they do get closer to the realities of frontline care than other safety systems. They still produce negotiated, acceptable accounts, subject to the many interdisciplinary tensions that characterise ward work. Glossing over these complexities may reduce staff engagement with these systems, if they are unable to recognise themselves or their practice in how the systems are implemented and managed.[56] Nonetheless, these prospective surveillance strategies – through which system flaws are mitigated as they become apparent – suggest a viable route to continuous safety improvement. They foster improvement not only by making soft data intelligible to healthcare managers, but by supporting frontline staff to make incremental changes in their daily work – a goal for learning healthcare organisations.

For peer review only

Box 1: Examples of service investment and organisational change arising from PCTS

Cost-neutral re-alignment of porter provision for radiology, doubling the in-patient flow for X-rays;

Re-evaluation of phlebotomy service provision, ultimately generating a £20,000 investment in an expanded support service;

Additional phlebotomy training for healthcare assistants, delivered internally;

Additional sonographer in echocardiography;

Relocation of intravenous infusion and nasogastric feeding pumps to a central equipment library;

Development of electronic referrals and electronic reporting for medical specialty consultations, using existing electronic health record;

£5,000 investment in ketone meters for management of diabetic ketoacidosis.

Contributions:

Study design: SP, SA, TA, NS, SJL, IB. Study implementation and data collection: SA, SP, SJL, IB. Analysis: SA, SP, NS. All authors contributed to, read and approved the final manuscript. Dr Pannick had full access to all of the data in the study and takes responsibility for the integrity of the data.

Financial support:

This paper represents independent research supported by the National Institute for Health Research (NIHR) Imperial Patient Safety Translational Research Centre, Imperial College Healthcare Charity (Grant GG14\1022), and West Middlesex University Hospital NHS Trust. NS' research is supported by the NIHR Collaboration for Leadership in Applied Health Research and Care South London at King's College Hospital NHS Foundation Trust. NS is a member of King's Improvement Science, which is part of the NIHR CLAHRC South London and comprises a specialist team of improvement scientists and senior researchers based at King's College London. Its work is funded by King's Health Partners (Guy's and St Thomas' NHS Foundation Trust, King's College Hospital NHS Foundation Trust, King's College London and South London and Maudsley NHS Foundation Trust), Guy's and St Thomas' Charity, the Maudsley Charity and the Health Foundation. NS is the director of London Training & Safety Solutions Ltd, which delivers team assessment and training to hospitals on a consultancy basis. No funding source had any role in the design and conduct of the study; collection,

For peer review only

management, analysis or interpretation of the data; or preparation, review or approval of the manuscript. The views expressed are those of the authors and not necessarily those of the NHS, the NIHR or the Department of Health.

Data sharing:

The qualitative data cannot be shared without identifying participants.

For peer review only

REFERENCES

1. World Health Organisation. Patient safety. 2014. http://www.who.int/features/factfiles/patient_safety/en/. Accessed 27th March 2016.
2. de Vries EN, Ramrattan MA, Smorenburg SM, et al. The incidence and nature of in-hospital adverse events: a systematic review. Qual Saf Health Care 2008;**17**(3):216-23.
3. Wong BM, Dyal S, Etchells EE, et al. Application of a trigger tool in near real time to inform quality improvement activities: a prospective study in a general medicine ward. BMJ Qual Saf 2015;**24**(4):272-81.
4. Macrae C. Early warnings, weak signals and learning from healthcare disasters. BMJ Qual Saf 2014;**23**(6):440-5.
5. Kirkup B. The Report of the Morecambe Bay Investigation. London: The Stationery Office, 2015.
6. Parand A, Burnett S, Benn J, et al. The disparity of frontline clinical staff and managers' perceptions of a quality and patient safety initiative. J Eval Clin Pract 2011;**17**(6):1184-90.
7. Dixon-Woods M, Baker R, Charles K, et al. Culture and behaviour in the English National Health Service: overview of lessons from a large multimethod study. BMJ Qual Saf 2014;**23**(2):106-15.
8. Martin GP, McKee L, Dixon-Woods M. Beyond metrics? Utilizing 'soft intelligence' for healthcare quality and safety. Soc Sci Med 2015;**142**:19-26.

9. Andrews LB, Stocking C, Krizek T, et al. An alternative strategy for studying adverse events in medical care. Lancet 1997;**349**(9048):309-13.

10. Dixon-Woods M, Suokas A, Pitchforth E, et al. An ethnographic study of classifying and accounting for risk at the sharp end of medical wards. Soc Sci Med 2009;**69**(3):362-9.

11. Lamba AR, Linn K, Fletcher KE. Identifying patient safety problems during team rounds: an ethnographic study. BMJ Qual Saf 2014;**23**(8):667-9.

12. Vincent C, Burnett S, Carthey J. The measurement and monitoring of safety. London: Health Foundation, 2013.

13. Tucker AL, Edmondson AC. Why hospitals don't learn from failures: Organizational and psychological dynamics that inhibit system change. California Management Review 2003;**45**(2):55-72.

14. Jeffs L, Berta W, Lingard L, et al. Learning from near misses: from quick fixes to closing off the Swiss-cheese holes. BMJ Qual Saf 2012;**21**(4):287-94.

15. Tucker AL. An empirical study of system improvement by frontline employees in hospital units. Manufacturing & Service Operations Management 2007;**9**(4):492-505.

16. Singer SJ, Gaba DM, Falwell A, et al. Patient safety climate in 92 US hospitals: differences by work area and discipline. Med Care 2009;**47**(1):23-31.

17. Tucker AL, Singer SJ, Hayes JE, et al. Front-line staff perspectives on opportunities for improving the safety and efficiency of hospital work systems. Health Serv Res 2008;**43**(5 Pt 2):1807-29.

18. Keogh B. Review into the quality of care and treatment provided by 14 hospital trusts in England. London: NHS, 2013. http://www.nhs.uk/NHSEngland/bruce-keogh-review/Documents/outcomes/keogh-review-final-report.pdf.

19. National Advisory Group on the Safety of Patients in England. A Promise to Learn - a Commitment to Act. London: Department of Health, 2013.

For peer review only

20. Pannick S, Beveridge I, Wachter RM, et al. Improving the quality and safety of care on the medical ward: A review and synthesis of the evidence base. Eur J Intern Med 2014;**25**(10):874-87.

21. Hogan H, Healey F, Neale G, et al. Preventable deaths due to problems in care in English acute hospitals: a retrospective case record review study. BMJ Qual Saf 2012;**21**(9):737-45.

22. Kennedy TJ, Regehr G, Baker GR, et al. Preserving professional credibility: grounded theory study of medical trainees' requests for clinical support. BMJ 2009;**338**:b128.

23. Pannick S, Beveridge I, Ashrafian H, et al. A stepped wedge, cluster controlled trial of an intervention to improve safety and quality on medical wards: the HEADS-UP study protocol. BMJ Open 2015;**5**(6):e007510.

24. Pannick S, Beveridge I, Athanasiou T, et al. Prospective clinical team surveillance improves safety climate, incident reporting and patient outcomes. A cluster controlled stepped wedge trial. Journal of Hospital Medicine 2016;**11 (suppl 1)**.

25. Moore GF, Audrey S, Barker M, et al. Process evaluation of complex interventions: Medical Research Council guidance. BMJ 2015;**350**:h1258.

26. Bate P. Context is Everything. Perspectives on Context. Health Foundation, 2014.

27. McGinity R, Salokangas M. Introduction: 'embedded research' as an approach into academia for emerging researchers. Management in Education 2014;**28**(1):3-5.

28. Vindrola-Padros C, Pape T, Utley M, et al. The role of embedded research in quality improvement: a narrative review. BMJ Qual Saf 2016;10.1136/bmjqs-2015-004877.

29. McMullen H, Griffiths C, Leber W, et al. Explaining high and low performers in complex intervention trials: a new model based on diffusion of innovations theory. Trials 2015;**16**:242.

30. Shaw SE, Russell J, Parsons W, et al. The view from nowhere? How think tanks work to shape health policy. Critical Policy Studies 2014;**9**(1):58-77.

For peer review only

31. Watson TJ. Ethnography, Reality, and Truth: The Vital Need for Studies of 'How Things Work' in Organizations and Management. Journal of Management Studies 2011;**48**(1):202-17.

32. Morgan DL. *Focus groups as qualitative research*. 2nd ed. ed. London: Sage; 1997.

33. Braun V, Clarke V. Using thematic analysis in psychology. Qualitative Research in Psychology 2006;**3**(2):77-101.

34. Birken SA, Lee SY, Weiner BJ. Uncovering middle managers' role in healthcare innovation implementation. Implement Sci 2012;**7**:28.

35. Buchanan D. Pure plays and hybrids: Acute trust management profile and capacity. Journal of Health Services Research & Policy 2013;**18**(2):90-97.

36. Tajfel H, Turner JC. An integrative theory of intergroup conflict. In: Austin WG, Worchel S, eds. The Social psychology of intergroup relations. Monterey, CA: Brooks/Cole, 1979.

37. van Os A, de Gilder D, van Dyck C, et al. Responses to professional identity threat: Identity management strategies in incident narratives of health care professionals. J Health Organ Manag 2015;**29**(7):1011-28.

38. Dixon-Woods M, McNicol S, Martin G. Ten challenges in improving quality in healthcare: lessons from the Health Foundation's programme evaluations and relevant literature. BMJ Qual Saf 2012;**21**(10):876-84.

39. Martin GP, Leslie M, Minion J, et al. Between surveillance and subjectification: professionals and the governance of quality and patient safety in English hospitals. Soc Sci Med 2013;**99**:80-8.

40. Brady PW, Muething S, Kotagal U, et al. Improving situation awareness to reduce unrecognized clinical deterioration and serious safety events. Pediatrics 2013;**131**(1):e298-308.

41. Vaughan D. *The Challenger launch decision: risky technology, culture, and deviance at NASA*. Chicago: University of Chicago Press; 1996.

42. Macrae C. The problem with incident reporting. BMJ Qual Saf 2016;**25**(2):71-5.

For peer review only

43. Mitchell I, Schuster A, Smith K, et al. Patient safety incident reporting: a qualitative study of thoughts and perceptions of experts 15 years after 'To Err is Human'. BMJ Qual Saf 2016;**25**(2):92-9.

44. Pannick S, Sevdalis N, Athanasiou T. Beyond clinical engagement: a pragmatic model for quality improvement interventions, aligning clinical and managerial priorities. BMJ Qual Saf 2016;**25**:716-25.

45. Dixon-Woods M, Martin G, Tarrant C, et al. Safer Clinical Systems: evaluation findings. London: The Health Foundation, 2014.

46. Forster AJ, Worthington JR, Hawken S, et al. Using prospective clinical surveillance to identify adverse events in hospital. BMJ Qual Saf 2011;**20**(9):756-63.

47. White WA, Kennedy K, Belgum HS, et al. The Well-Defined Pediatric ICU: Active Surveillance Using Nonmedical Personnel to Capture Less Serious Safety Events. Jt Comm J Qual Patient Saf 2015;**41**(12):550-60.

48. Waring JJ. Constructing and re-constructing narratives of patient safety. Soc Sci Med 2009;**69**(12):1722-31.

49. Degeling P, Maxwell S, Kennedy J, et al. Medicine, management, and modernisation: a "danse macabre"? BMJ 2003;**326**(7390):649-52.

50. Hewett DG, Watson BM, Gallois C, et al. Intergroup communication between hospital doctors: implications for quality of patient care. Soc Sci Med 2009;**69**(12):1732-40.

51. Rowland P, Kitto S. Patient safety and professional discourses: implications for interprofessionalism. J Interprof Care 2014;**28**(4):331-8.

52. Hooper P, Kocman D, Carr S, et al. Junior doctors' views on reporting concerns about patient safety: a qualitative study. Postgrad Med J 2015;**91**(1075):251-6.

53. Goldenhar LM, Brady PW, Sutcliffe KM, et al. Huddling for high reliability and situation awareness. BMJ Qual Saf 2013;**22**(11):899-906.

For peer review only

54. Rotter T, Kinsman L, James E, et al. Clinical pathways: effects on professional practice, patient outcomes, length of stay and hospital costs. Cochrane Database Syst Rev 2010;10.1002/14651858.CD006632.pub2(3):CD006632.

55. Thomas EJ. The future of measuring patient safety: prospective clinical surveillance. BMJ Qual Saf 2015;**24**(4):244-5.

56. Iedema R. New approaches to researching patient safety. Social Science & Medicine 2009;**69**(12):1701-04.

For peer review only

BMJ Open

Translating concerns into action: a detailed qualitative evaluation of an interdisciplinary intervention on medical wards

Journal:	*BMJ Open*
Manuscript ID	bmjopen-2016-014401.R1
Article Type:	Research
Date Submitted by the Author:	17-Jan-2017
Complete List of Authors:	Pannick, Samuel; Imperial College London, NIHR Imperial Patient Safety Translational Research Centre<br>Archer, Stephanie; Imperial College London, NIHR Imperial Patient Safety Translational Research Centre<br>Johnston, Maximillian; Imperial College London,<br>Beveridge, Iain; West Middlesex University Hospital NHS Trust, Department of Medicine<br>Long, Susannah; Imperial College London, Centre for Patient Safety and Service Quality<br>Athanasiou, Thanos; Imperial College London, Surgery and Cancer<br>Sevdalis, Nick; King's College London,
**Primary Subject Heading**:	Health services research
Secondary Subject Heading:	Health services research, Medical management, Qualitative research
Keywords:	Quality in health care < HEALTH SERVICES ADMINISTRATION & MANAGEMENT, interdisciplinary teams, medical ward, prospective surveillance

SCHOLARONE™
Manuscripts

Translating concerns into action: a detailed qualitative evaluation of an interdisciplinary intervention on medical wards

Samuel Pannick,[1] Stephanie Archer,[1] Maximilian J Johnston,[1] Iain Beveridge,[2]
Susannah J Long,[1,3] Thanos Athanasiou,[4] & Nick Sevdalis[5]

[1] NIHR Imperial Patient Safety Translational Research Centre, Imperial College London
[2] West Middlesex University Hospital, Isleworth, UK
[3] Imperial College Healthcare NHS Trust, London, UK
[4] Department of Surgery & Cancer, Imperial College London, UK
[5] Centre for Implementation Science, King's College London

Corresponding author:
Dr Sam Pannick, MA MB BS MRCP
Room 503 Medical School Building, Norfolk Place
London W2 1NY
United Kingdom
s.pannick@imperial.ac.uk; + (44) 207 3543149

Key words: patient safety; healthcare quality; interdisciplinary teams; medical ward; prospective surveillance

Abstract length: 279 words

Manuscript length: 4971 words

Additional content: Box 1

For peer review only

ABSTRACT

Objectives

To understand how frontline reports of day-to-day care failings might be better translated into improvement.

Design

Qualitative evaluation of an interdisciplinary team intervention to capitalise on the frontline experience of care delivery. Prospective clinical team surveillance (PCTS) involved structured interdisciplinary briefings to capture challenges in care delivery, facilitated organisational escalation of the issues they identified, and feedback. Eighteen months of ethnography and two focus groups were conducted with staff taking part in a trial of PCTS.

Results

PCTS fostered psychological safety – a confidence that the team would not embarrass or punish those who speak up. This was complemented by a hard edge of accountability, whereby team members would regulate their own behaviour in anticipation of future briefings. Frontline concerns were triaged to managers, or resolved autonomously by ward teams, reversing what had been well-established normalisations of deviance. Junior clinicians found a degree of catharsis in airing their concerns, and their teams became more proactive in addressing improvement opportunities. PCTS generated tangible organisational changes, and enabled managers to make a convincing case for investment. However, briefings were constrained by the need to preserve professional credibility, and the relative comfort afforded by the avoidance of accountability. At higher organisational levels, frontline concerns were subject to competition with other priorities, and their resolution was limited by the scale of the challenges they described.

For peer review only

Conclusions

Prospective safety strategies relying on staff-volunteered data do approximate the realities of frontline care, but still produce acceptable, negotiated accounts, subject to the many interdisciplinary tensions that characterise ward work. Nonetheless, they give managers access to these accounts, and support frontline staff to make incremental changes in their daily work. These are goals for learning healthcare organisations.

Trial registration

ISRCTN 34806867

STRENGTHS AND LIMITATIONS OF THIS STUDY

Qualitative evaluations of complex interventions are recommended by the Medical Research Council.

This study identified the mechanisms to be replicated if further PCTS implementation efforts are to prove successful.

The evaluation highlighted tensions within efforts to capitalise on the frontline experience of care delivery; previous reports had characterised frontline data collection as a more objective exercise.

Selection bias may reduce the internal validity of our findings: there was disproportionate access to staff who engaged with the intervention.

As a single-site study, the generalisability of the results may be limited.

For peer review only

INTRODUCTION

Around 10% of hospitalised patients suffer preventable harms.[1,2] Many are the result of persistent organisational failings, either deliberately ignored, or to which the organisation has become blind.[3] Frontline staff have a unique insight into the reality of failings in day-to-day care, identifying safety and quality concerns in the course of their routine work.[4-7] Their concerns – revealing uncomfortable institutional fallibilities – are not reflected in high-level organisational metrics. Rather, frontline insights into everyday processes are 'soft data', adding a nuanced understanding that can build more holistic improvement strategies.[8]

Senior healthcare leaders acknowledge the importance of this frontline knowledge – yet those same leaders are loath to spend time and resources pursuing staff concerns that might ultimately prove to be trivial.[9] Few safety campaigns focus on the tribulations of frontline workers.[10,11] Instead, staff typically devise their own workarounds for operational problems, producing temporary fixes. This 'first-order' problem solving is effective in the moment – but it does little to prevent each problem's recurrence, and organisational processes go unchanged.[12-14]

'Prospective clinical surveillance' is one mechanism for improving care delivery from a frontline perspective. Embedded observers (or visiting facilitators) work with frontline staff to record their experiences of care delivery and its consequences, with a structure to support data capture.[2,15,16] Documenting potentially flawed care processes and adverse events, prospective clinical surveillance can produce detailed, unit-level performance assessments in near-real time.[17] It is 'prospective' in two respects. First, it proactively seeks out staff concerns, rather than waiting for staff to volunteer them. Second, it can identify care deficiencies that have not yet led to patient harm. However, its effectiveness as an improvement strategy is unclear. Whether prospective clinical surveillance translates staff concerns into tangible action, and how exactly it might do so, have not previously been explored.

For peer review only

Here, we investigate the qualitative impact of prospective clinical *team* surveillance (PCTS), a novel extension of this technique. PCTS had three components: (i) structured interdisciplinary briefings to capture frontline clinical and administrative challenges; (ii) facilitated organisational escalation of the issues they identified; and (iii) feedback. PCTS was evaluated in a cluster controlled trial on medical wards, involving seven interdisciplinary teams from two London (UK) hospitals.[18] High fidelity PCTS reduced excess length of stay; it also improved teamwork and safety climates, and incident reporting.[19] In this study, we seek to describe the intervention's effects as understood and enacted by participating staff.

METHODS

Intervention

The intervention is described in detail in the published protocol.[18] In brief, structured briefings reviewed the events of the previous shift, identifying clinical and administrative challenges, and concluding with a plan to resolve or escalate those concerns. A structured pro forma (HEADS-UP; Hospital Event Analysis Describing Significant Unanticipated Problems) focused on the problems most commonly identified on medical wards.[20] After a short period of supervision, ward teams led their own briefings autonomously. A single facilitator helped teams advance the issues raised in their briefings, and provided follow-up and feedback to stakeholders throughout the organisation. Briefings were known locally as 'HEADS-UP briefings'. The autonomous team briefings were intended to be a sustainable local alternative to the facilitator-led data capture described in previous reports.[2,16]

Process evaluation

This study was grounded in the Medical Research Council framework for process evaluations of complex interventions.[21] Qualitative methods identify the complex causal pathways and unexpected

For peer review only

mechanisms of impact by which these interventions produce change. Ethnography and focus groups are recommended for this type of investigation:[22] they are used here to evaluate the intervention at the hospital site most heavily involved in the primary study.

Embedded research and auto-ethnography

The programme lead was an embedded researcher at the institution contributing six of the seven study wards. This position is defined as 'work[ing] inside [a] host organisation as [a] member of staff, while also maintaining an affiliation with an academic institution… conduct[ing] research studies… which respond to the needs of the organisation, and accord with its unique context and culture'.[23] Relationships between staff and the researcher are important: the researcher is seen both to be part of the team and contributing to the research capacity of the host organisation.[24] Fieldnotes, typed contemporaneously as soon as was practicable, recorded personal exchanges with staff, changes arising from PCTS, governance proceedings, and reflections on the broader context. These 'auto-ethnographic' insights have been widely used in organisational case study research, providing broad accounts of culture and practice.[25-27]

Focus groups

Semi-structured focus groups were undertaken in July 2015. The topics of interest were specific attitudes, feelings and beliefs that would best be revealed through social interactions between group participants.[28] The topic guide explored PCTS as a tool, as well as the implementation process that introduced it. To orientate them, participants were asked to discuss existing systems for identifying team concerns and improving patient care. They then reflected on their experiences of using PCTS. Questions and follow-up probes scrutinised team-wide involvement in PCTS; whether it affected perceptions of the ward's safety and quality; how it was used in staff interactions; and whether changes had been made as a result. Topic guide questions were reviewed and piloted within the research team, and with researchers in healthcare quality and safety who had not been

For peer review only - http://bmjopen.bmj.com/site/about/guidelines.xhtml

involved with the study previously. The focus groups were advertised in clinical areas, by email, and in person; all ward staff were invited to attend. Certain staff groups were purposively targeted to ensure that key stakeholders (junior doctors, senior nurses, and service managers) were represented. Focus groups lasted approximately two hours, facilitated by experienced qualitative researchers (N.S., M.J.J, S.A.; also Dr Louise Hull [Research Fellow] and Ms Tayana Soukup [Research Assistant]), using the topic guide flexibly in view of the different roles and experiences within the two groups. Focus group discussions were digitally recorded in the hospital's medical education centre, and then professionally transcribed.

Focus group transcripts and fieldnotes were managed with NVivo software (QSR International, Doncaster, Australia). Two researchers (S.P., S.A.) read and re-read the source material, adopting an inductive (theory-generating) approach.[29] This type of analysis is a flexible research tool, generating a rich and detailed account of a complex data set. It can be applied to focus groups as well as other qualitative data,[30-32] allowing thematic integration in a single analysis. Each researcher coded transcripts individually, generating an initial coding frame, which was then discussed and refined. The transcripts were coded again, before a group of higher order themes was agreed. Field notes were searched for evidence to support or contradict the evidence from the focus groups, and for themes not covered independently by focus group discussions. Themes were inspected against the broader literature to inform their interpretation. The study was approved by research and development authorities at participating institutions as a quality improvement programme. Focus group participants gave their signed consent. The primary study was registered with the ISRCTN registry (ISRCTN34806867).

RESULTS

Fifteen staff participated in the focus groups: three junior doctors (foundation doctors, in their first two years of medical practice), eight senior ward nurses, and four non-clinical managers.

For peer review only

Participants represented each of the clinical divisions that had taken part in the study at this hospital (care of the elderly, acute medicine, respiratory medicine, and gastroenterology). Focus group data were supplemented by 44 pages of auto-ethnographic fieldnotes, documenting the 18-month period in which links were established with clinicians; clinical and non-clinical managers; and senior hospital leaders.

Our findings are organised around four themes. First, we describe the shared team beliefs and ethos established by PCTS. Second, we discuss how teams used the programme to triage ward problems more effectively. Third, we identify how individuals' practice changed as a result of their participation. Finally, we note how PCTS altered team and organisational approaches to improvement, and the tangible changes that came about.

1. PCTS established psychological safety, with a hard edge of accountability

By the end of the study period, all staff groups had participated in – and led – HEADS-UP briefings:

> '[Consultants] contribute as much as anyone else […] so their voice is being heard, because they suffer from the same frustrations as all of us.' *(Service manager)*

> 'The physio[therapists] or the OTs [occupational therapists] are also flagging things up, that's how we found it useful. […] The discharges that did not happen, anyone [who] deteriorated […] we all learn from what went wrong.' *(Senior nurse)*

Perhaps because different staff groups were perceived as partners in them, the briefings formed a psychologically safe environment in which problems could be discussed openly, without fear of

For peer review only - http://bmjopen.bmj.com/site/about/guidelines.xhtml

retribution. Team psychological safety does not imply undue permissiveness or unrelenting cheeriness, but a confidence that the team will not embarrass or punish someone for speaking up:

> 'It was more of a constructive exercise, where everyone is then wanting the same outcome, so it wasn't like, "Oh this didn't happen, I am angry at you", it was more like we are all actually in the same team […] You all have the same end point and I think that is why it is quite safe to bring it up, because it is not confrontational, and it is not personal against someone, it is just what needs to be done.' *(Foundation doctor)*

The style and timeliness of PCTS feedback also contributed to a sense that this was a non-judgemental forum for team learning:

> 'There were a few teaching sessions where […] we went through things like ECGs [electrocardiographs] that were missed, and then you had a collective opportunity to think […] It was a very good plot for learning, saying this happened and let's do some learning and try and prevent it happening again. That is quite helpful.' *(Foundation doctor)*

This psychological safety was not limitless: there were still boundaries around what could be discussed. Although the briefings did prompt teams to consider positive notes from the previous shift, they rarely did so. Reflections often highlighted the overall management of patient flow, rather than specific actions or diagnostic processes that others could emulate. This reflected a reluctance to delve too deeply into any one team member's performance. Professional identities were protected by assigning problems to other departments, acknowledging procedural complications as foreseeable, or linking delays to under-staffing. In this way, perceived threats to professional identity were largely deflected by a projection of blame onto other groups, or attributed to circumstances beyond the team's control.

For peer review only

More introspective teams, with senior support and increasing psychological safety, did record issues like diagnostic errors, in which they had played a more overt role. However, even in those teams – where the briefings were implemented with greatest fidelity – there could be disputes as to whether certain problems were really problems at all:

> 'The senior nurse disagrees with medical teams' perceptions that the site moves [moving patients from one ward to another] were inappropriate. She thinks doctors expect patients to remain on the acute medical unit even when they are stable… She felt an inadequate handover perpetuated their concerns.' *(Fieldnotes)*

Thus, whilst psychological safety was an important factor in helping staff speak up about their concerns, certain topics remained off-limits, and psychological safety did not guarantee agreement about what were reasonable concerns. These are important limitations:

> 'I think it's more [than] just raising the issue. […] Give me a more detailed timeframe, resolution, how do you resolve it? Are you going to get back to me because it's important? And what will I expect?' *(Senior nurse)*

Where they were most effective, the briefings maintained a 'hard edge': team members regulated their own behaviour, knowing that their actions (or inactions) might be flagged up at the next briefing, or in feedback. Junior doctors and nurses were conscious of this internalised discipline (panopticism), acknowledging how it changed their own behaviour and how they could use it to their advantage. A self-reinforcing virtuous cycle emerged, with participation, accountability, and feedback:

For peer review only - http://bmjopen.bmj.com/site/about/guidelines.xhtml

For peer review only

‘The group was more aware that if you perhaps missed something like that, it may be brought up later at a HEADS-UP meeting. […] It made you more accountable.’ *(Foundation doctor)*

‘It certainly works for the VTEs [venous thromboembolism assessments, completed by junior doctors, often at the behest of nursing staff]. There are days that we will have eight or 10 VTEs [to do], so if I […] give the list to the doctor, the following day […] there will be one or two, because they know […] it will be mentioned again, with the consultant.’ *(Senior nurse)*

‘You get more feedback, so you raise concern[s], and then maybe the next day the same issue would crop up, and you actually then get feedback on what they are doing about it, or who they have escalated to.’ *(Foundation doctor)*

As we will now describe, airing and recording concerns in this environment helped triage those problems, directing them back to frontline teams or onwards to their managers.

2. Rapid resolution and meaningful managerial follow-up

To explain their perceptions of PCTS, focus group participants drew comparisons with the existing processes for identifying and resolving ward-level problems. The hospital’s online incident reporting system was considered the formal mechanism by which problems were recorded. The system was not easy to use for this purpose, and there were few attempts to persistently report recurrent problems. As a result, commonplace issues were no longer even considered abnormal – a materialisation of normalised deviance, where staff become so desensitised to deviant practice that it no longer feels wrong:

For peer review only

'Some things staff have got so used to that they don't [report] it. So it's just become common practice.' *(Clinical risk manager)*

Managers too were frustrated by the incident reporting system. Reports did not necessarily illuminate what was happening at the frontline. Moreover, long delays in processing those incident reports meant that even relatively simple problems were not reviewed for many weeks. As a result, reports that described patient harm were prioritised: where no harm had occurred, little was done. Service managers were aware that they paid attention to only the most serious incidents:

'You could go down there [to the ward] and you could see people working very, very hard and you could also sense a lot of frustrations […] but you weren't getting to what those frustrations were, and what those issues were.' *(Service manager)*

'It has got to that point of being a SI [serious incident, an adverse event with particularly grave consequences] before it is then addressed and taught and learnt from.' *(Service manager)*

In contrast, PCTS helped identify a route for more rapid resolution of practical problems. It provided an acceptable mechanism for staff to log issues into which they had immediate insight. This was a materialisation of second-order problem solving, staff finding a way to bring about a more lasting solution to each issue they encountered. Where issues could not be resolved within the ward team, managers were presented with clear, actionable topics to address. Those managers were then better prepared to handle incipient risks to service quality:

'[PCTS] highlighted little things, that could be sorted out quite quickly. It made sure that somebody was allocated to deal with that on the day – or did you follow that through, did

For peer review only

that get sorted out? So everything benefits, rather than just going on and on and on […] Things get sorted out quicker.' *(Senior nurse)*

'What I like about it is [that] it is instant and it is thematic very quickly, you understand what the issues are […] Here is an opportunity to dump them [your daily issues] and suddenly a picture forms out of it […] And not just here, but there, there, there and there […] And it hasn't yet created a significant incident, but clearly there is risk associated with it.' *(Service manager)*

PCTS therefore brought about faster resolution of safety and quality issues through a combination of mechanisms. First, increasing self-monitoring within ward teams helped anticipate and mitigate potential problems. Second, senior ward staff at the daily briefings were more quickly aware of issues they could resolve, without having to trawl through a backlog of outstanding incident reports. Third, facilitation drew out thematic problems across departments, bringing them to the attention of middle managers who had struggled to understand the difficulties experienced by their staff. Fourth, the clinical impact of these problems was made clearer to senior executives, who were motivated by these novel frontline narratives to pursue change:

'PCTS findings informed the discussion in the Trust morbidity and mortality meeting, chaired by the chief executive, highlighting areas which needed faster examination and transformation, and bringing genuinely new information... Many of these concerns had not been formally addressed either in incident reports or top-down initiatives... It also highlighted new targets for improvement.' *(Fieldnotes)*

However, not all managers were equally enthused. Where PCTS had not provided data that appeared directly useful for them, middle managers did less to hold their service areas accountable

For peer review only - http://bmjopen.bmj.com/site/about/guidelines.xhtml

for briefing implementation. Board members questioned how best to detect a meaningful signal amongst the noise of staff reporting (whether through formal incident reports or PCTS). At times, managers seemed nihilistic about the possibility of improvement, or questioned whether their frontline teams truly understood the problems they had reported:

> 'We have two chances of getting [this issue resolved] – fat chance and no chance! We are monitoring [this] on a daily basis but […] the situation is far from ideal and we have escalated up the chain – not that it has helped.' *(Support service manager – Fieldnotes)*

> 'I would like to get some feedback on [these issues] first […] Currently they are only perception[s], and following investigation it may be that the risk changes, or the issue is solely around communication of processes already in place.' *(General manager – Fieldnotes)*

Still, the combination of increased frontline efficiency and managerial involvement (at least in some areas) led to a more general sense that things had changed for the better:

> '[PCTS] would make it clear what was actually deficient. And then I think things did start to change, and you did see the feedback coming through […] So you could definitely see the evolution of it.' *(Foundation doctor)*

> 'There [are] also the […] day-to-day things within the team that start running better, and I think you do start seeing those, but it is harder to put a specific, "This was raised, this was done, there was an outcome." It is a more […] general change as part of [PCTS].' *(Foundation doctor)*

For peer review only

With a structure in place to coordinate frontline teams and their managers, individual staff found their own practice changing, with unexpected personal benefits. These will now be discussed in more detail.

3. Changing individuals: reversing normalised deviance, and catharsis

PCTS prompted staff to proactively address issues they may have previously ignored. This was, in effect, a reversal of the normalised deviance that had become so ingrained:

> 'You wouldn't have identified that [issue] necessarily, because it is just part of your daily […] life in the NHS, but when it is put like this [with PCTS], it is highlighted, you have got an opportunity to really do something about it.' *(Service manager)*

> 'This particular patient [has been] awaiting echo[cardiography] for the last three days […] [I] would now question why is it still not done […] When I wasn't doing [the briefings], I wasn't seeing the importance […] [Now] I tell the Matron, or I tell my Bed Manager […] or I go there myself […] I go to the department and ask them, "This is what's happening, this is what I need."' *(Senior nurse)*

Having made those problems visible once again, the information shared in the HEADS-UP briefings would be used to actively coordinate plans and decisions for the next shift. Aware of pressures on their colleagues, and cognisant that problems were occurring repeatedly, individuals would change their routines:

For peer review only

> ‘I tell the physio[therapist] and the OT [occupational therapist]: “These are your priorities for today, these are your priorities for tomorrow.” So they already have a plan […] Like I said, it gives you the structure that you need.’ *(Senior nurse)*

> ‘The group as a whole became more “present” to [what] happens maybe a couple of times in a week […] For example if the nursing staff are short […] just having that in your mind and thinking “No, we need to get all these blood tests done now”, you would appreciate how the service could be best run. I think that made it more efficient.’ *(Foundation doctor)*

The improved information sharing, interdisciplinary coordination, and efforts to evenly distribute workload produced a more supportive team climate. In this atmosphere, the briefings could become unexpectedly cathartic, giving a voice to junior clinicians to air their personal worries, as well as their clinical concerns:

> ‘You felt like you had got a lot of your chest by doing it […] The next day you could say, “These are the things that went wrong yesterday”, and now I can clear that and start again.’ *(Foundation doctor)*

> ‘[It] makes you feel more heard, because otherwise you are just venting your concern, but you don’t know if it has been actioned in any way. And then you can provide the objective evidence to move forward, to change something.’ *(Foundation doctor)*

The combination of changes in team ethos, triage, and individual practice ultimately provoked a new approach to improvement, and a degree of organisational development.

For peer review only

4. Changing teams and the organisation

Contrary to some leaders' fears, PCTS did not reduce the volume of formal incident reports. Rather, it provoked new reports, and altered how those reports were addressed. Yet the translation of briefing-recorded incidents into online reports was imperfect, not least because there was no agreed list of high priority events to be recorded:

> 'Antibiotics had not been given […] The junior doctors had not seen the procedure report specifying they were needed. No incident report was done, even though [the registrar] specifically asked if one should be completed.' *(Fieldnotes)*

When incidents were reported, PCTS briefings allowed incident investigators to start to collect relevant information. The problems that had provoked the incident were typically captured by the briefing, giving the investigator (often the nurse in charge) a head start in understanding what had happened:

> `The people involved are already there, they are already able to explain to you, "This is what actually happened from our end." […] It's easier for you to do your incident [investigation] report already because as the investigator you don't have to call everyone.' *(Senior nurse)*

The emphasis on clinical teams' accountability also led to a subtler change in incident management. Teams took responsibility for issues that were actually within their power to change, rather than awaiting permission or hoping that the problems would be resolved elsewhere:

> 'Very often before it gets to me, it has been dealt with and sorted out by the teams themselves […] With HEADS-UP and [incident reporting] together, there is a different

For peer review only

approach: […] "We have discussed it, we have taken on the responsibility of this, we have done as much of this as we can, but here is the point where it has to be escalated and we want to make sure something is done about it, and this is our methodology for doing it." That is how it feels to me, anyway.' *(Service manager)*

At multiple levels within the organisation, it was clear that this programme had revealed hidden gaps in practice. PCTS proved useful even on wards where teams were already thought to be functioning well:

`There is definitely time to do it, there is definitely a need to do it, but […] you only know that once you have done it.' *(Service manager)*

'I don't think we would have known, unless we had done it, that there was a gap there.' *(Senior nurse)*

Similarly, in safety and governance meetings, PCTS findings highlighted new areas for improvement, many of which had not been addressed in incident reports or top-down initiatives. Those findings enabled managers to make a convincing case for investment, with numerous specific, tangible changes driven by PCTS during the study period [box 1]. These changes were described as 'quick wins' by senior managers, with clinicians agreeing that they were at least a relatively rapid organisational response:

'It empowers me then as manager for that area to go forward with a business case with the evidence to say we need additional resource, this is the implications of it […] And it works, so we have an additional [cardiac] sonographer […] I think we saw very quickly a reduction in delays once that was sorted.' *(Service manager)*

For peer review only - http://bmjopen.bmj.com/site/about/guidelines.xhtml

However, internal investment was not necessarily targeted specifically to participating teams. For example, expansion of the phlebotomy service (identified through PCTS as no longer meeting patient needs) primarily benefited non-participant wards, which were deemed by senior management to be more in need of support. Nor was progress guaranteed by repeated ward-level reporting. Changes to structures or processes were more likely when there was an associated financial target; another organisational incentive aligning with the proposed improvement; and when clinicians and managers agreed the need for change.

Additionally, some of the concerns raised by frontline staff simply could not be addressed within the organisation. Poor staffing levels, for example, were felt keenly, but reflected a broader national challenge. Creative workarounds and mitigation plans could only go so far in addressing a fundamental shortfall in staff numbers. This was frustrating for the teams completing the briefings. PCTS also identified issues like unsafe inter-hospital transfers, whose resolution would have required close collaboration within a network of hospitals. With many other competing priorities, a local improvement effort was no guarantor of the necessary cooperative drive for change.

DISCUSSION

PCTS established a basis of team psychological safety, married with a hard edge of accountability. Ward teams triaged recurrent problems to their managers, or resolved them autonomously, reversing normalised deviance through non-judgemental facilitation and feedback. Junior clinicians found a degree of catharsis, and teams became more proactive in addressing improvement opportunities. The soft intelligence provided through PCTS proved effective in generating tangible organisational change – albeit unpredictably, dependent on the hospital's other priorities and incentives. At the ward level, briefings were limited by the need to preserve professional credibility, a lack of interdisciplinary consensus about the nature of the problems they were seeing, and the

For peer review only - http://bmjopen.bmj.com/site/about/guidelines.xhtml

relative comfort afforded by the avoidance of accountability. At higher organisational levels, frontline concerns were subject to competition with other priorities, and resolution was limited by the full scale of the challenges they described.

To our knowledge, this report is the first qualitative analysis of how this type of interdisciplinary intervention might generate improved outcomes. Previous observational studies of prospective clinical surveillance had portrayed staff as objective data recorders: how they might actively shape, frame, and mitigate safety concerns was not considered. Our current study therefore offers two novel contributions. First, it identifies the mechanisms to be replicated if other PCTS implementation efforts are to prove successful. Second, it highlights the innate tensions within these efforts to capitalise on the frontline experience of care delivery.

There are multiple sources of friction in organisational learning. For example, when reporting their concerns, staff portray 'others' as threatening patient safety, rather than jeopardising their own credibility.[33,34] Disputes about appropriate care are common, not least because there are multiple interpretations of what the right course of action *should* have been.[35,36] These distinctions are lost as concerns are escalated through an organisation: rich narratives about patient safety events are progressively sanitised of their context.[34] Thus, whilst senior executives are encouraged to obtain 'first-hand knowledge of the system',[37] there is no single unproblematic version of the reality of frontline care – however we seek to understand it. One study of executive walkrounds (visits to the 'shop floor') suggested that these visits, whether announced or unexpected, necessarily revealed partial, biased accounts of what was going on.[38] Here too, we found that our structured programme to characterise frontline care was subject to interdisciplinary (and clinico-managerial) disputes and tensions. Prospective clinical surveillance has been described as 'the future of measuring patient safety'.[17] We would now argue that these systems are as value-laden and subjective as any other attempt to understand frontline care delivery.

For peer review only - http://bmjopen.bmj.com/site/about/guidelines.xhtml

Limitations of this study include our disproportionate access to the teams and individuals who engaged with PCTS. These enthusiasts may have had a more positive view of the programme than the wider group of eligible clinicians and managers. Selection bias has hampered mixed methods evaluations of other quality improvement interventions.[39] We attempted to counteract this bias with focus group facilitators who had not been directly involved in delivering the PCTS programme, and searched for conflicting opinions in the group transcripts and fieldnotes. Our results may also have been influenced by social desirability bias – participants giving the acceptable 'right' answers rather than revealing their true thoughts – and may not generalise fully to other settings. Nonetheless, our findings are consistent with, and build on, the existing understanding of frontline safety engagement.[40] Strengths of the study include the theoretically-informed analysis of an emergent safety surveillance strategy, and dual methods allowing themes to be integrated. The embedded research model produced good access to managers as well as frontline staff: often, there is a trade-off between the two. Importantly, the study identified mechanisms by which frontline concerns can be used productively, rather than disappearing into an organisational 'black hole'[13] from which they generate no change or learning.

CONCLUSIONS

Healthcare systems can only improve with a detailed operational understanding of how care actually takes place.[41] Prospective clinical surveillance strategies offer a route to this understanding. These strategies are not merely novel measurement tools, as they have been described[15-17] – even if they do get closer to the realities of frontline care than other safety systems. They still produce negotiated, acceptable accounts, subject to the many interdisciplinary tensions that characterise ward work. Nonetheless, prospective surveillance strategies – through which system flaws are mitigated as they become apparent – facilitate continuous safety improvement. They foster improvement not only by making soft data intelligible to healthcare managers, but by supporting

For peer review only - http://bmjopen.bmj.com/site/about/guidelines.xhtml

frontline staff to make incremental changes in their daily work – a goal for learning healthcare organisations.

Box 1: Examples of service investment and organisational change arising from PCTS
Cost-neutral re-alignment of porter provision for radiology, doubling the in-patient flow for X-rays;
Re-evaluation of phlebotomy service provision, ultimately generating a £20,000 investment in an expanded support service;
Additional phlebotomy training for healthcare assistants, delivered internally;
Additional sonographer in echocardiography;
Relocation of intravenous infusion and nasogastric feeding pumps to a central equipment library;
Development of electronic referrals and electronic reporting for medical specialty consultations, using existing electronic health record;
£5,000 investment in ketone meters for management of diabetic ketoacidosis.

Contributions:

Study design: SP, SA, MJJ, TA, NS, SJL, IB. Study implementation and data collection: SA, SP, MJJ, SJL, IB. Analysis: SA, SP, NS. All authors contributed to, read and approved the final manuscript. Dr Pannick had full access to all of the data in the study and takes responsibility for the integrity of the data.

Acknowledgements:

We are grateful to Dr Louise Hull and Ms Tayana Soukup for their help in facilitating the focus groups.

Financial support:

This paper represents independent research supported by the National Institute for Health Research (NIHR) Imperial Patient Safety Translational Research Centre, Imperial College Healthcare Charity (Grant GG14\1022), and West Middlesex University Hospital NHS Trust. NS' research is supported by the NIHR Collaboration for Leadership in Applied Health Research and Care South London at King's

For peer review only - http://bmjopen.bmj.com/site/about/guidelines.xhtml

College Hospital NHS Foundation Trust. NS is a member of King's Improvement Science, which is part of the NIHR CLAHRC South London and comprises a specialist team of improvement scientists and senior researchers based at King's College London. Its work is funded by King's Health Partners (Guy's and St Thomas' NHS Foundation Trust, King's College Hospital NHS Foundation Trust, King's College London and South London and Maudsley NHS Foundation Trust), Guy's and St Thomas' Charity, the Maudsley Charity and the Health Foundation. NS is the director of London Training & Safety Solutions Ltd, which delivers team assessment and training to hospitals on a consultancy basis. No funding source had any role in the design and conduct of the study; collection, management, analysis or interpretation of the data; or preparation, review or approval of the manuscript. The views expressed are those of the authors and not necessarily those of the NHS, the NIHR or the Department of Health.

Data sharing:

The qualitative data cannot be shared without identifying participants.

For peer review only

REFERENCES

1. de Vries EN, Ramrattan MA, Smorenburg SM, et al. The incidence and nature of in-hospital adverse events: a systematic review. Qual Saf Health Care 2008;**17**(3):216-23.

2. Wong BM, Dyal S, Etchells EE, et al. Application of a trigger tool in near real time to inform quality improvement activities: a prospective study in a general medicine ward. BMJ Qual Saf 2015;**24**(4):272-81.

3. Macrae C. Early warnings, weak signals and learning from healthcare disasters. BMJ Qual Saf 2014;**23**(6):440-5.

4. Andrews LB, Stocking C, Krizek T, et al. An alternative strategy for studying adverse events in medical care. Lancet 1997;**349**(9048):309-13.

5. Dixon-Woods M, Suokas A, Pitchforth E, et al. An ethnographic study of classifying and accounting for risk at the sharp end of medical wards. Soc Sci Med 2009;**69**(3):362-9.

6. Lamba AR, Linn K, Fletcher KE. Identifying patient safety problems during team rounds: an ethnographic study. BMJ Qual Saf 2014;**23**(8):667-9.

7. Vincent C, Burnett S, Carthey J. The measurement and monitoring of safety. London: Health Foundation, 2013.

8. Dixon-Woods M, Baker R, Charles K, et al. Culture and behaviour in the English National Health Service: overview of lessons from a large multimethod study. BMJ Qual Saf 2014;**23**(2):106-15.

9. Martin GP, McKee L, Dixon-Woods M. Beyond metrics? Utilizing 'soft intelligence' for healthcare quality and safety. Soc Sci Med 2015;**142**:19-26.

10. Singer SJ, Gaba DM, Falwell A, et al. Patient safety climate in 92 US hospitals: differences by work area and discipline. Med Care 2009;**47**(1):23-31.

11. Tucker AL, Singer SJ, Hayes JE, et al. Front-line staff perspectives on opportunities for improving the safety and efficiency of hospital work systems. Health Serv Res 2008;**43**(5 Pt 2):1807-29.

12. Tucker AL, Edmondson AC. Why hospitals don't learn from failures: Organizational and psychological dynamics that inhibit system change. California Management Review 2003;**45**(2):55-72.

13. Jeffs L, Berta W, Lingard L, et al. Learning from near misses: from quick fixes to closing off the Swiss-cheese holes. BMJ Qual Saf 2012;**21**(4):287-94.

14. Tucker AL. An empirical study of system improvement by frontline employees in hospital units. Manufacturing & Service Operations Management 2007;**9**(4):492-505.

15. Forster AJ, Worthington JR, Hawken S, et al. Using prospective clinical surveillance to identify adverse events in hospital. BMJ Qual Saf 2011;**20**(9):756-63.

16. White WA, Kennedy K, Belgum HS, et al. The Well-Defined Pediatric ICU: Active Surveillance Using Nonmedical Personnel to Capture Less Serious Safety Events. Jt Comm J Qual Patient Saf 2015;**41**(12):550-60.

17. Thomas EJ. The future of measuring patient safety: prospective clinical surveillance. BMJ Qual Saf 2015;**24**(4):244-5.

For peer review only

18. Pannick S, Beveridge I, Ashrafian H, et al. A stepped wedge, cluster controlled trial of an intervention to improve safety and quality on medical wards: the HEADS-UP study protocol. BMJ Open 2015;**5**(6):e007510.

19. Pannick S, Beveridge I, Athanasiou T, et al. Prospective clinical team surveillance improves safety climate, incident reporting and patient outcomes. A cluster controlled stepped wedge trial. Journal of Hospital Medicine 2016;**11 (suppl 1)**.

20. Lubberding S, Zwaan L, Timmermans DR, et al. The nature and causes of unintended events reported at 10 internal medicine departments. J Patient Saf 2011;**7**(4):224-31.

21. Moore GF, Audrey S, Barker M, et al. Process evaluation of complex interventions: Medical Research Council guidance. BMJ 2015;**350**:h1258.

22. Bate P. Context is Everything. Perspectives on Context. Health Foundation, 2014.

23. McGinity R, Salokangas M. Introduction: 'embedded research' as an approach into academia for emerging researchers. Management in Education 2014;**28**(1):3-5.

24. Vindrola-Padros C, Pape T, Utley M, et al. The role of embedded research in quality improvement: a narrative review. BMJ Qual Saf 2016;10.1136/bmjqs-2015-004877.

25. McMullen H, Griffiths C, Leber W, et al. Explaining high and low performers in complex intervention trials: a new model based on diffusion of innovations theory. Trials 2015;**16**:242.

26. Shaw SE, Russell J, Parsons W, et al. The view from nowhere? How think tanks work to shape health policy. Critical Policy Studies 2014;**9**(1):58-77.

27. Watson TJ. Ethnography, Reality, and Truth: The Vital Need for Studies of 'How Things Work' in Organizations and Management. Journal of Management Studies 2011;**48**(1):202-17.

28. Morgan DL. *Focus groups as qualitative research*. 2nd ed. ed. London: Sage; 1997.

29. Braun V, Clarke V. Using thematic analysis in psychology. Qualitative Research in Psychology 2006;**3**(2):77-101.

For peer review only

30. Morrison LG, Yardley L. What infection control measures will people carry out to reduce transmission of pandemic influenza? A focus group study. BMC Public Health 2009;**9**:258.

31. Totman J, Hundt GL, Wearn E, et al. Factors affecting staff morale on inpatient mental health wards in England: a qualitative investigation. BMC Psychiatry 2011;**11**:68.

32. Barnett J, Vasileiou K, Djemil F, et al. Understanding innovators' experiences of barriers and facilitators in implementation and diffusion of healthcare service innovations: a qualitative study. BMC Health Serv Res 2011;**11**:342.

33. Kennedy TJ, Regehr G, Baker GR, et al. Preserving professional credibility: grounded theory study of medical trainees' requests for clinical support. BMJ 2009;**338**:b128.

34. Waring JJ. Constructing and re-constructing narratives of patient safety. Soc Sci Med 2009;**69**(12):1722-31.

35. Degeling P, Maxwell S, Kennedy J, et al. Medicine, management, and modernisation: a "danse macabre"? BMJ 2003;**326**(7390):649-52.

36. Hewett DG, Watson BM, Gallois C, et al. Intergroup communication between hospital doctors: implications for quality of patient care. Soc Sci Med 2009;**69**(12):1732-40.

37. National Advisory Group on the Safety of Patients in England. A Promise to Learn - a Commitment to Act. London: Department of Health, 2013.

38. Martin G, Ozieranski P, Willars J, et al. Walkrounds in practice: corrupting or enhancing a quality improvement intervention? A qualitative study. Jt Comm J Qual Patient Saf 2014;**40**(7):303-10.

39. Goldenhar LM, Brady PW, Sutcliffe KM, et al. Huddling for high reliability and situation awareness. BMJ Qual Saf 2013;**22**(11):899-906.

40. Mitchell I, Schuster A, Smith K, et al. Patient safety incident reporting: a qualitative study of thoughts and perceptions of experts 15 years after 'To Err is Human'. BMJ Qual Saf 2016;**25**(2):92-9.

For peer review only

41. Bohmer RM. The Hard Work of Health Care Transformation. N Engl J Med 2016;**375**(8):709-11.

For peer review only

Coreq checklist

1. Personal characteristics:

Interviewer / facilitator – page 7
Credentials – page 1
Occupation – pages 1, 7
Gender – page 1, 7
Experience – pages 1, 7

2. Relationship with participants:

Relationship – pages 6, 21
Participant knowledge – page 7
Interviewer characteristics – page 6

3. Theoretical framework:

Methodological orientation – page 7

4. Participant selection:

Sampling – page 7
Method of approach – page 7
Sample size – page 7
Non-participation – N/A

5. Setting

Data collection – page 7
Non-participants – N/A
Sample description – pages 7,8

6. Data collection

Interview guide – page 6
Repeat interviews – N/A
Audio recording – page 7
Field notes – page 6
Duration – page 7
Data saturation – N/A
Transcripts – N/A

7. Analysis

Data coders – page 7
Coding tree – N/A

For peer review only

Derivation of themes – page 7
Software – page 7
Participant checking – N/A

8. Reporting

Quotations presented – pages 8-19
Data and findings – pages 8-19
Clarity of major themes – pages 8-19
Clarity of minor themes – pages 8-19

For peer review only
